# Bluetongue Virus Infections in Cattle Herds of Manabí Province of Ecuador

**DOI:** 10.3390/pathogens10111445

**Published:** 2021-11-06

**Authors:** Euclides De la Torre, Nixon Moreira, Claude Saegerman, Kris De Clercq, María Salinas, Alex Maldonado, David Jarrín, María Sol Vaca, Silvia Pachacama, Jorge Espinoza, Hipatia Delgado, Maritza Barrera

**Affiliations:** 1 Animal Diagnostic Laboratory, Agencia Ecuatoriana para el Control Fito y Zoosanitario (AGROCALIDAD), Quito 170184, Ecuador; euclides.delatorre@agrocalidad.gob.ec (E.D.l.T.); maria.salinas@agrocalidad.gob.ec (M.S.); ruben.maldonado@agrocalidad.gob.ec (A.M.); jorge.espinoza@agrocalidad.gob.ec (J.E.); 2 Dirección Distrital Manabí, Agencia Ecuatoriana para el Control Fito y Zoosanitario (AGROCALIDAD), Portoviejo 130105, Ecuador; 3 Research Unit in Epidemiology and Risk Analysis Applied to Veterinary Sciences (UREAR ULiège), Fundamental and Applied Research for Animal and Health (FARAH) Center, Department of Infectious and Parasitic Diseases, Faculty of Veterinary Medicine, University of Liège, 4000 Liege, Belgium; 4 Unit of Exotic and Particular Diseases, Scientific Directorate Infectious Diseases in Animals, Sciensano, Groeselenberg 99, 1180 Brussels, Belgium; Kris.DeClercq@sciensano.be; 5 Molecular Biology Laboratory, Agencia Ecuatoriana para el Control Fito y Zoosanitario (AGROCALIDAD), Quito 170184, Ecuador; david.jarrin@agrocalidad.gob.ec (D.J.); maria.vaca@agrocalidad.gob.ec (M.S.V.); silvia.pachacama@agrocalidad.gob.ec (S.P.); 6 Veterinary Department, Faculty of Veterinay Sciences, Universidad Técnica de Manabí, Portoviejo 130105, Ecuador; hipatia.delgado@utm.edu.ec

**Keywords:** bluetongue, BTV, cattle, PCR, competitive ELISA, Ecuador, Manabí

## Abstract

Bluetongue (BT) is a viral disease transmitted by *Culicoides* (Diptera: *Ceratopogonidae*) to domestic and wild ruminants. Infections in cattle are mainly subclinical, but severe necrotic and hemorrhagic illness and death may occur depending on the strain of the virus and other factors; cattle act as a reservoir for the virus. Although the Ecuadorian coast has climatic conditions that favor the presence of the vector, there are few serologic or virologic BTV studies available. Manabí is a coastal province in which livestock farming is mostly implemented in the northern part. We conducted two studies to assess, for the first time, the presence of active BTV infections in Manabí province. We collected 430 serum samples from 38 randomly selected farms between March and July 2019 to perform BTV competitive ELISA. In addition, six seropositive farms were selected to place eight sentinel BTV-naive calves. All these calves were blood sampled and the presence of BTV RNA and antibodies was tested for by RT-PCR and competitive ELISA, respectively, once a week for 6–8 weeks until seroconversion was evidenced. A high individual seroprevalence (99%) was obtained, and all investigated farms had BTV seropositive animals. All sentinel calves became BTV viremic and seroconverted. The first viremia appeared after 2–5 weeks from arrival at the farm; they seroconverted 1–3 weeks later. We demonstrate for the first time that there is a high level of BTV circulation north of Manabí, with active infections on these farms. Integrated control strategies such as hygienic measures on farms to reduce midge populations would be advisable for the owners as mitigation measures.

## 1. Introduction

Bluetongue (BT) is an infectious, non-contagious disease caused by bluetongue virus (BTV), which is transmitted to domestic and wild ruminants by vectors [1]. Currently, 27 serotypes have been widely recognized, but nine other putative serotypes, have been proposed [2,3,4,5,6]. Depending on the BTV serotype, the clinical manifestations of the disease vary between species, from being unapparent in the vast majority of infected animals to fatal in a proportion of sheep, cattle, deer, and other wild ruminants; cattle act as a reservoir of the virus [1,7,8].

The infection of BTV is included on the OIE list of notifiable diseases that infect multiple species [9]. The major adverse impact of BTV infection in many regions of the world lies in the economic losses due to restrictions on the international movement of ruminant livestock and germplasm [10].

The biting midge of several species of Culicoides (Diptera: Ceratopogonidae) are the biological vectors for BTV [10,11,12]. The species involved in BTV transmission are variable with the geographic area, but *Culicoides sonorensis* is the principal vector of BTV throughout much of North America, whereas *Culicoides insignis* is the major vector of BTV in the Caribbean Basin, Central and South America, and in Florida and the Gulf Coast region of the south eastern United States of America (reviewed by [13]) and is directly associated with the transmission of BTV between ruminant and non-ruminant natural hosts. However, species of Culicoides present in Ecuador could be more diverse [14]. The virus is present from latitude 35° S to 53° N, which corresponds broadly to the tropical and subtropical range, but with northern extension [15,16]. The climatic conditions in these areas favor the survival of the vector and BTV infections. 

In Ecuador, there are four geographical zones, each with a different height above sea level and different climatic conditions. They are: the coast to the east, the central region with the Andes Mountains, the Amazon Rainforest to the west, and the Galápagos Islands. Manabí is a coastal province with a tropical climate, but it is influenced by marine currents, and in the rainy season (December–May), the climate is humid, with the formation of numerous natural deposits of rainwater that favor the environmental conditions for the life cycle of culicoides. More than 30% of the province’s livestock production is concentrated in the Chone municipality, north of Manabí province. These herds are characterized by dual-purpose cattle in extensive and semi-intensive conditions. Sheep and goat farming are not common.

BTV infection has been reported in South America, in areas with climatic conditions suitable for the life cycle of the vector (reviewed by [17]). Almost all countries have serological evidence of the presence of BTV, but only Brazil and Argentina have BTV isolation [17,18,19]. Lobato et al. [18] has indicated the presence of other endemic diseases with similar clinical patterns as a probable cause of unreported clinical cases of BTV. Recently, serologic surveys have been conducted in sheep and cattle in Brazil [19,20,21,22,23]; outbreaks in sheep have been reported [23]. Perú, which shares a common border with Ecuador, recently reported BTV infections in sheep [24].

The first serological evidence of the circulation of BTV in Ecuador was reported by [25]; however, perhaps because the disease occurs in cattle with mild clinical signs, the presence of this viral disease has not been reported in the country. However, Verdesoto et al. [26] found bovines with a positive diagnosis of bluetongue infection by PCR and ELISA. These BTV positive samples came from the Andean region of Tandapi, the Amazonian region (Cotundo), and from a slaughterhouse in Santo Domingo, on the border of coastal and Andean regions. However, all sera were found seronegative for BTV in the 2014 survey, conducted on farms of three of the islands of the Galápagos Islands (part of the Ecuadorian territory) [27]. In addition, no publication was found on BTV in sheep and goats in Ecuador. Sheep are uncommon in the Manabí costal area (they are more common in higher altitude).

With the aim of assessing, for the first time, the circulation of the virus and the presence of active BTV infections in Manabí province, we conducted the present investigation in two parts: a cross-sectional serologic survey and a longitudinal study with sentinel calves in six selected seropositive farms from the Chone area.

## 2. Results

### 2.1. Cross-Sectional Survey

In the cross-sectional survey, all farms were seropositive for BTV according to cELISA. Out of 430 cattle sera tested with cELISA, 426 were seropositive for BTV-specific antibodies, representing an apparent animal seroprevalence of 99% (Table 1). Based on the sensitivity and specificity of the cELISA and their apparent prevalence estimated above, the true prevalence was estimated using the Rogan-Gladen formula [28] to be between 99.08 and 100%, corresponding to a high level of BTV circulation.

### 2.2. Longitudinal Study

In the longitudinal study, all sentinel calves were viremic and subsequently seroconverted (Table 2). The first viremia appeared between week two and five from the arrival of animals at the farm. Animals seroconverted between week three and eight post-arrival. Five sentinels (numbers: 1, 2, 3, 5, and 7) exhibited seroconversion between one and three weeks after the first PCR positive result. The viremia and seroconversion confirm that the calves had been productively infected with BTV, even though we did not attempt virus isolation. 

Sentinel calves 4, 6, and 8 were both PCR- and ELISA-positive at the same sampling time (Table 2). Calf 4 was in San Antonio B, calf 6 was in Ricaurte, and calf 8 was in Boyacá, and therefore in different farms and places. Calves 4 and 6 were fed with cow substitute milk. Calf 8 was fed with balanced milk. BTV infection and BTV seroconversion became clear during the week between the two samplings. 

**Figure 1:**
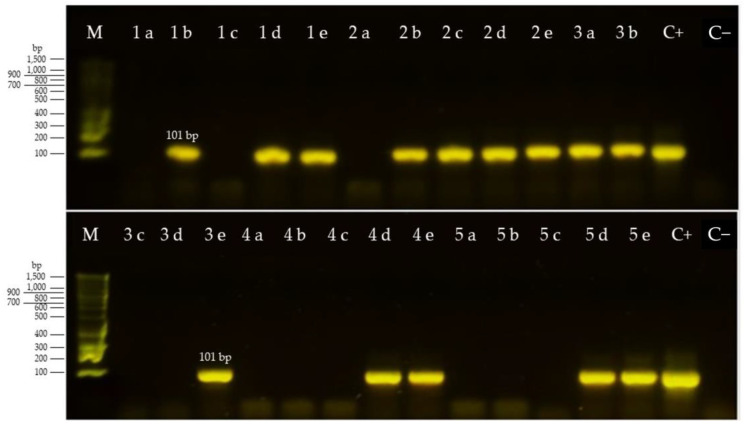
Agarose electrophoresis of the second PCR product for different dates of sampling of sentinel calves. Legend: M = molecular size marker (100 pb); 1 = calf 4; 2 = calf 5; 3 = calf 6; 4 = calf 7; 5 = calf 8; dates are a = 25/6/2019; b = 3/7/2019; c = 9/7/2019; d = 15/7/2019; e = 23/7/2019; C+ = reference strain BTV-2; C− = water (negative control).

The PCR reaction was BTV-specific and the products of the second round of the nested RT-PCR had the expected size (Figure 1). The electrophoresis of the products of the second round of the nested RT-PCR of the extracted RNA from the blood-EDTA samples corresponding to three sentinel calves (4, 5, and 6) at weeks 2, 3, 4, and 5 is presented in Figure 1.

No clinical signs or increased mortality were reported by the owners during the cross-sectional survey. The sentinel calves had a retarded growth or died after the end of the longitudinal experiment.

## 3. Discussion

A high true animal prevalence was evidenced in the survey area. It is probably due to the peak of the seasonal vector-activity during the sampling period. In addition, all negative sentinel calves became viremic and subsequently seroconverted. Indeed, as we hypothesized, Manabí province has the environmental conditions for the development of BTV infections. Our results have shown a wide circulation of the virus among cattle of the investigated farms, similar to [26], who obtained such high values of seropositivity (98.9%) in 295 samples of cattle without clinical signs of infection from two farms in the Amazonian and Andean parishes and a slaughterhouse in Santo Domingo. Santo Domingo is located near the slope of the Andes Mountains and has different climate conditions than Manabí. The two BTVs isolated in the Andean parish (Alluriquin) from Santo Domingo province were different serotypes (BTV-9 and BTV-13) and BTV-18 was isolated from one sample from the Amazonian parish of Cotundo. The circulating serotype in Manabí, with such a different climate and geographic relief, may be another, so the isolation and further serotyping of Manabí’s BTV is very necessary.

The first BTV reports in America were made in the 1980s in the Caribbean and Central America region, where the disease was found to be endemic. There is viral activity every year in these regions, resulting in subclinical infections and a high prevalence of antibodies [12,29]. Gibbs et al. [30] found an overall BTV seroprevalence of 70% in bovines from seven countries in the Caribbean and from two in South America. A BTV prevalence of 84% was found in French Guyana [31]. In Cuba, with a similar climate in the rainy season as Manabí, the BTV prevalence reported was 99.7% [32]. More recently, a 100%BTV prevalence in cattle from seven regions of Sao Paulo was obtained by [23].

Comparing our results with the first report of 10.3% [25], the circulation of BTV has increased and has become endemic in Ecuador. The causes of such an increase could be related to the livestock development in the country, and especially to the area under study (Chone). Cattle from Paraguay have been imported, looking for genetic improvement. Furthermore, the presence of the Culicoides vector and the absence of control measures for the vector and BTV could explain the high proportion of seropositive animals. Nevertheless, the ecologic causes that could favor the presence of Culicoides in the Manabí province have not been investigated. There is only one report about the Culicoides species in Ecuador [33]; however, this report was from Tiputine (Orellana province), which is a very humid tropical region with an Amazonia climate, different from the Manabí coastal climate, which has a dry period and a humid period. In another study, Mosquera Bolaños [34] found only one *C. Insignis* specimen among the 326 females tested that was positive for BTV in RT-qPCR in the locality of Cotundo. Further studies are needed to confirm this first finding and to establish more properly the presence of other candidate vector species.

The active epidemiological longitudinal study that we carried out in the six selected farms by introducing sentinel calves allowed us to demonstrate that there are active infections in these farms. Although it was not the objective of the experiment to demonstrate the presence of virus-loaded female Culicoides, this presence was indirectly demonstrated because susceptible cattle placed on these farms became viremic and further seroconverted.

Sentinel calves 4, 6, and 8 were both PCR- and ELISA-positive at the same sampling time (Table 2). Calves 4 and 6 were fed by cow substitute milk. In this case, it was not possible to exclude a BTV RNA transmission by colostrum, as evidenced in the past by [35], because colostrum was unfortunately not tested. However, Calf 8 was not fed cow substitute milk, and BTV infection and BTV seroconversion became evident during the week in between the two samplings. This is plausible because BTV-RNA could be detected as early as 3 days after being bitten by female Culicoides [36] and the c-ELISA is able to detect antibodies as early as 7 days after infection [37,38].

Finally, no clinical signs or unusual increased mortality were reported by the owners during the cross-sectional survey. However, naïve calves involved in the longitudinal study experienced retarded growth or died after the end of the experiment. Due to the variability of clinical outcomes induced by different strains of BTV [39,40], future study of the virulence of circulating Ecuadorian BTV strains in cattle is necessary.

## 4. Conclusions

The cross-sectional survey allowed us to obtain preliminary epidemiological information on the presence and circulation of BTV in Ecuador. The active epidemiological study using animal sentinels could help to implement a reliable national program to determine the areas of Ecuador where BTV is endemic and to collect the BTV circulating strains for further serotyping and phylogenetic study. This information is essential for the implementation of a control program based on vaccination. 

## 5. Materials and Methods

### 5.1. Cross-Sectional Survey

**Figure 2:**
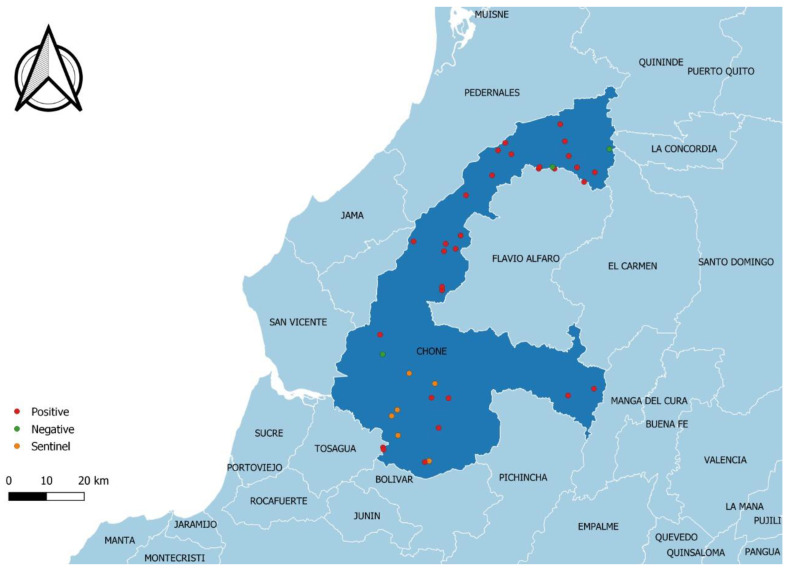
Geographic location of the farms selected in the municipality of Chone (province of Manabí). Red dots indicate farms with 100% of BTV seroreactors; green dots indicate farms selected with one or two negative BTV competitive ELISA results; orange dots indicate farms where sentinel calves were placed.

A descriptive cross-sectional study to estimate the frequency of BTV seropositivity was conducted between March and July 2019, in which 38 farms were randomly selected from nine parishes of Chone in the north of Manabí province (Figure 2). The cattle population of Chone was 241,910 bovines older than 6 months of age and was distributed over 6650 farms listed in the foot and mouth disease vaccination register created in 2018.

In this study, the total number of farms randomly selected was calculated assuming a 95% level of confidence, an expected prevalence of 90% with an acceptable error of 10%, i.e., 35 farms plus 10% for possible non-participation of some farmers and rounded at 38 farms. The total number of animals randomly sampled and tested by BTV cELISA was calculated assuming a 90% level of confidence and an expected prevalence of 50%, with an acceptable error of 4%, i.e., 423 animals, rounded to 430 bovines.

### 5.2. Longitudinal Study with Animal Sentinels

Six of the farms investigated in the serological survey were selected as locations to place sentinel calves (orange dots in Figure 2) [41]. These farms were located in four rural parishes (i.e., Canuto, San Antonio, Boyaca, and Ricaurte). The inclusion criteria were based on the presence of BTV seropositive animals in the herd with a higher morbidity rate compared to other farms, and the consent of the owners. Eight crossbreed Holstein newborn calves (7–12 days of age) were chosen as animal sentinels and were bought from two milk farms situated at Machachi (with a dry and cold climate, where the Culicoides vector cannot survive). Previously, all these calves were sampled; all calves tested negative for BTV antibodies by c-ELISA. The calves were transferred to Chone in two groups and 1–2 calves were placed in each previously selected farm. The calves were fed with cow substitute milk or balanced milk (powder milk for calves, Josten-Milk (Imported from Holland) plus balanced feed for calves, (Procesadora Nacional de Alimentos C.A. Pronaca, Puembo, Quito, Ecuador)). Blood samples were taken from every calf each week using EDTA and tested by a pan BTV-PCR, and without anticoagulant to collect serum for the BTV c-ELISA for 6–8 weeks post-arrival, until seroconversion was evidenced.

### 5.3. Laboratory Analyses

BTV-competitive ELISA (cELISA): All serum samples from the cross-sectional survey and sentinel study were analyzed using a cELISA for the determination of specific antibodies to BTV (VMRD SA, Pullmann, WA, USA) [42,43,44]. This cELISA detects antibodies to BTV in ruminant sera. The sample serum BTV antibody inhibited the binding of horseradish peroxidase (HRP)-labelled BTV-specific monoclonal antibodies to BTV antigen coated on the plastic wells of the microtiter plate. Binding of the HRP-labelled monoclonal antibody conjugate is detected by the addition of enzyme substrate and quantified by subsequent colour product development. Strong colour development indicates little or no blockage of (HRP)-labelled monoclonal antibody binding and therefore the absence of BTV antibody in sample sera. Weak colour development due to the inhibition of the monoclonal antibody binding to the antigen on the solid phase indicates the presence of BTV antibodies in sample sera.

According to the producer, the sensitivity of BTV cELISA is 98.1% and the specificity is 97.74% (95% CI: 95.60–99.02). 

The percentage of inhibition (%I) was calculated using the following formula:

% I = 100 [1 − (Sample OD/NC OD)(1)

where I is the inhibition; OD is the optical density; NC is the negative control sample; and Sample is the tested sample.

The test was validated only if the mean of the NC produced an OD > 0.300 and <2.000 and the mean of the positive controls had an inhibition of ≥60%. The results were interpreted as positive or negative when a test sample produced ≥60% and <60% inhibition, respectively.

BTV-PCR test: The EDTA blood samples were sent refrigerated to the AGROCALIDAD laboratories (Quito, Ecuador). They were stored at −80 °C until the moment of analysis.

Blood samples corresponding to the sampling times before and after the seroconversion occurred were analyzed by reverse transcription-nested PCR (RT-nPCR) [45,46]. Total RNA purification of each sample was performed and subsequently analyzed by RT-nPCR to detect BTV RNA. For RNA purification, a High Pure RNA Purification Kit (Roche, Mannheim, Germany) was used. Furthermore, extracted RNA was analyzed by reverse transcription with Enzyme RT MLV (Promega, Madison, WI, USA), followed by nested PCR with the set of primers BTV-A, BTV-B, BTV-C, BTV-D [45,46] and a green Flexi PCR kit (PROMEGA, Madison, WI, USA). The PCR products were run on agarose electrophoresis stained with SyberSafe using the Bench Top 100pb MW marker (Promega, Madison, WI, USA) As a positive control, we used RNA extracted from a BTV-2 (Reference strain (ATCC^®^ VR-983™), obtained from Dizar Laboratories (Bogotá, Colombia) grown on BHK-21 cell cultures (ATCC^®^ CCL10™). The expected size of the final PCR product from the second n-PCR reaction is 101 bp [45].

### 5.4. Statistical Analyses

The exact binomial distribution was used to derive 95% confidence intervals (95% CI) of the BTV prevalence [47]. All analyses were performed using Stata SE 14.2 (StataCorp, College Station, TX, USA, 2015) and the alpha level was set to 0.05.

The animal true prevalence (TP) of BTV was estimated from the apparent prevalence (AP) calculated in this study using cELISA (i.e., the seroprevalence) and the individual diagnostic specificity (Sp) and sensitivity (Se) based on the producer claims, using the Rogan–Gladen formula [28]:

TP = (AP + Sp − 1)/(Se + Sp − 1)(2)

For the AP, Sp, and Se values of the different cELISA, a uniform variable was used, taking into account the extreme values of the 95% confidence interval. Stocastic modelling (1000 Monte Carlo simulations) was performed using @Risk 7.5.2 software (Palisade Corporation, Ithaca, NY, USA) to estimate the TP with a 95% CI using Equation (2).

## Figures and Tables

**Table 1:** Results of bluetongue virus serological cross-sectional survey using competitive ELISA in farms of different rural parishes of Chone in the north of Manabí province.

Rural Parishes	Number of Farms	Estimated Cattle Population	Total Number of Samples	ELISA (+)	ELISA (−)	Positive Rate (%)
Boyacá *	3	1073	52	51	1	98.1
Canuto *	5	677	68	67	1	98.5
Chibunga	15	1652	141	139	2	98.6
Chone	3	59	19	19	0	100
Convento	6	581	68	68	0	100
Eloy Alfaro	2	143	30	30	0	100
Ricaurte *	1	313	10	10	0	100
San Antonio *	2	276	16	16	0	100
Santa Rita	2	214	26	26	0	100
Total	38	4988	430	426	4	99.1

* The farms included in the longitudinal study were located from these rural parishes.

**Table 2:** Weeks post-arrival that the sentinel calves became viremic and seroconverted in the longitudinal study.

Farm	Canuto A		Canuto B		San Antonio A		San Antonio B				Ricaurte				Boyacá	
Calf No.	1		2		3		4		5		6		7		8	
Test	PCR	ELISA	PCR	ELISA	PCR	ELISA	PCR	ELISA	PCR	ELISA	PCR	ELISA	PCR	ELISA	PCR	ELISA
Weeks post arrival	5	8	3	4	6	7	5	5	2	4	3	3	3	5	5	5
Feed	Balanced milk *		Cow substitute milk		Balanced milk		Cow substitute milk		Cow substitute milk		Cow substitute milk		Cow substitute milk		Balanced milk	
Sampling date	First sampling 5 March 2019 Last sampling 6 May 2019						First sampling 18 June 2019 Last sampling 23 July 2019									
Arrival	26 February 2019						13 June 2019									

The farms in which each sentinel calf was placed were: calf 1—Canuto A; calf 2—Canuto B; calf 3—San Antonio A; calf 4—San Antonio B; calf 5—San Antonio B; calf 6—Ricaurte; calf 7—Ricaurte; calf 8—Boyacá. * The balanced milk was prepared by dissolving the powdered milk in warm water and adding the balanced food at proportions suggested by the manufacturer. Balanced food is constituted by corn, wheat, sorghum, soy, sunflower and/or cotton flours, banana flour, palm kernel flour or paste, palm heart flour, soybean paste, corn co-products, wheat co-products, rice co-products, brewery co-products, passion fruit co-products, palm co-products, palm oil and/or free fatty acids, protected fat, calcium carbonate, calcium phosphate, salt, sodium bicarbonate, cane molasses; vitamin A, D3, and E supplement; trace mineral supplement: manganese sulfate, zinc sulfate, copper sulfate, ferrous sulfate, sodium selenite, calcium iodate, cobalt carbonate, sublimated sulfur, carbamide, protected carbamide, methionine, monensin, lasalocid, antifungal (organic acids), enzymes, sequestrant, and antioxidant.

## Data Availability

Not applicable.
